# CAMTA1–PPP3CA–NFATc4 multi-protein complex mediates the resistance of colorectal cancer to oxaliplatin

**DOI:** 10.1038/s41420-022-00912-x

**Published:** 2022-03-24

**Authors:** Ruijun Pan, Zhou Zhang, Hongtao Jia, Junjun Ma, Chao Wu, Pei Xue, Wei Cai, Xiaoping Zhang, Jing Sun

**Affiliations:** 1 Department of General Surgery, Ruijin Hospital Shanghai Jiaotong University School of Medicine, Shanghai, China; 2 Shanghai Minimally Invasive Surgery Center, Shanghai, China; 3 Department of General Surgery, Shidong Hospital Yangpu District, Shidong Hospital, Yangpu District, Shanghai, China; 4 Department of Nuclear Medicine, Shanghai Tenth People’s Hospital, Tongji University, Shanghai, 200072 China; 5 Shanghai Center of Thyroid Diseases, Tongji University School of Medicine, Shanghai, 200072 China

**Keywords:** Cancer therapeutic resistance, Colon cancer

## Abstract

Colorectal cancer is a major contributor to the worldwide prevalence of cancer-related deaths. Metastasis and chemoresistance are the two main causes for colorectal cancer treatment failure, and thus, high mortality. Calmodulin-binding transcription activator 1 (CAMTA1) is involved in tumor growth and development, but its mechanisms of action in the development of colorectal cancer and chemoresistance are poorly understood. Here, we report that *Camta1* is a tumor suppressor. Immunohistochemical staining and western blotting analyses of normal and colorectal cancer tissues showed a significantly low expression of *Camta1* expression in colorectal cancer tissues, when compared to adjacent normal tissues. In functional in vitro experiments, we observed that *Camta1* overexpression significantly decreased the proliferation and invasion capacity of SW620 and SW480 cells, whereas *Camta1* knockdown displayed a significant increase in the proliferative and invasive ability of these cells. Subsequently, we examined the effects of *Camta1* overexpression and knockdown on the resistance of colorectal cancer cells to oxaliplatin, a common chemotherapeutic drug. Interestingly, the sensitivity of *Camta1*-overexpressed cells to oxaliplatin was increased, whereas that of *Camta1*-silenced cells to the same chemotherapeutic drug was decreased. Furthermore, *Camta1* knockdown upregulated nuclear factor of activated T cells, cytoplasmic 4 (*Nfatc4*) mRNA, and protein levels in colorectal cancer cells and downregulated the phosphorylated NFATc4 level. By contrast, *Nfatc4* knockdown reversed the resistance of colorectal cancer cells to oxaliplatin caused by *Camta1* knockdown. In addition, we show that protein phosphatase 3 catalytic subunit alpha (PPP3CA) is essential for the expression and phosphorylation of NFATc4 caused by *Camta1* knockdown, as well as the proliferation, invasion, and chemoresistance of colorectal cancer cells. We show that PPP3CA and CAMTA1 competitively bind to NFATc4, and *Camta1* knockdown promotes the dephosphorylation of PPP3CA and suppresses the phosphorylation of NFATc4. To verify the role of CAMTA1 in oxaliplatin resistance in colorectal cancer, we established a xenograft mouse model and show agreement between in vitro and in vivo results.

## Introduction

Cancer is a key cause of mortality among humans. As such, it is not surprising that the incidence and prevalence of colorectal cancer is gradually increasing worldwide [1, 2]. Colorectal cancer is defined as cancer of the large intestine, the final region of the digestive tract, and cancer of the rectum, the last six inches of the large intestine, and it generally affects older individuals (i.e., those aged over 50 years), although it can also occur in younger individuals [3]. In addition to advanced age, other risk factors for colorectal cancer are a poor diet (i.e., low fiber, high fat), obesity, a history of smoking and/or alcohol use, a non-active lifestyle, a history of noncancerous colon polyps, and a (familial) history of colorectal cancer [4–8]. Colorectal cancer is often diagnosed at late stages, as most individuals do not have symptoms until the disease has progressed and possibly metastasized, and symptoms include bloody stools, bloating, abdominal pain, a bowel habit change (i.e., diarrhea and/or constipation), and fatigue. For these reasons, regular screenings, which include colonoscopy, are important. Currently, the treatment modalities for colorectal cancer are radiation, chemotherapy, and/or surgery, and the first-line chemotherapeutic drugs are 5-fluorouracil and oxaliplatin, although other targeted therapies are also available [8, 9].

Recently, several studies have provided evidence for the existence of a novel family of calmodulin-binding transcription activators (CAMTAs) that participate in transcriptional events by binding to specific cis-regulatory elements enriched in the promoters of specific genes. Calmodulin binding transcription activator 1 (CAMTA1), a key member of this transcription factor family, is also a tumor suppressor, inhibiting the growth of a variety of cancers, including glioma and colon cancer [10]. Furthermore, CAMTA1 is a highly-sensitive biomarker that has been used in the differential diagnosis of hepatic epithelioid hemangioendothelioma and angiosarcoma, two vascular tumor types, and low *CAMTA1* expression has been associated with poor prognosis in colorectal cancer [11]. Here, we investigate the role of CAMTA1 in colorectal cancer by manipulating its expression through overexpression and knockdown and examining how these changes affect the resistance of colorectal cancer cells to oxaliplatin. We hypothesize that *CAMTA1* knockdown can promote oxaliplatin resistance, thereby facilitating colorectal cancer growth.

Nuclear factor of activated T cells, cytoplasmic 4 (NFATc4) is a member of another transcription factor family, the NFAT family, whose activation is regulated by calcineurin, a Ca^++^- and calmodulin-dependent serine/threonine protein phosphatase, and its role in a variety of cancers has been established [12–14]. The NFAT family also functions in the immune system, specifically in triggering the immune response, and till date, five NFAT isoforms have been discovered (i.e., NFATc1–5). Interestingly, increased NFATc4 expression has been identified to correlate with poorer prognosis among cancer patients, similar to CAMTA1, and increased NAFTc4 activity mediates resistance to cisplatin, another platinum-based chemotherapeutic drug, in ovarian cancer, indicating that abnormal NFATc4 expression leads to treatment challenges. Furthermore, degradation of protein phosphatase 3 catalytic subunit alpha (PPP3CA), the catalytic subunit of calcineurin, may suppress tumor growth and metastasis, further supporting the role of the NFAT signaling pathway in carcinogenesis [15], although additional functional studies are required to understand the functions of NAFTc4 and PPP3CA in colorectal cancer.

Here, we report the function of CAMTA1 in colorectal cancer. We demonstrate that *CAMTA1* expression in colorectal cancer tissues is significantly lower than that in normal colorectal tissues from humans. In in vitro experiments, we show that the sensitivity of *CAMTA1*-overexpressed colorectal cancer cells to oxaliplatin was increased, whereas that of *CAMTA1*-silenced cells to oxaliplatin was decreased, indicating that CAMTA1 promotes the death of colorectal cancer cells exposed to this drug. Interestingly, *NFATC4* knockdown can reverse the chemoresistance of *CAMTA1*-silenced colorectal cancer cells to oxaliplatin, and this cellular event is partly mediated by an interacting protein, PPP3CA, which together with CAMTA1, competitively binds to NFATc4. Lastly, we use a xenograft mouse model to verify the role of CAMTA1 in oxaliplatin resistance in colorectal cancer and show agreement between in vitro and in vivo findings.

## Results

### CAMTA1 expression is decreased in colorectal cancer in humans

**Fig. 1:**
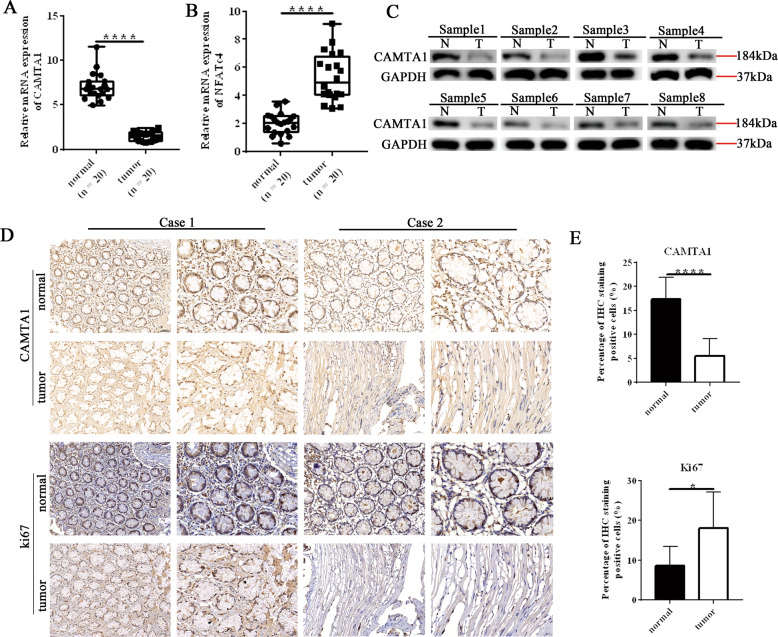
*CAMTA1* expression is decreased in colorectal cancer in humans. **A**, **B** qPCR analysis of *CAMTA1* (**A**) and *NFATc4* (**B**) mRNA levels in normal (*n* = 20) and colorectal cancer tissues (*n* = 20). **C** Western blot analysis of the CAMTA1 protein level in colorectal cancer (T) and paired adjacent normal (N) tissues from ten patients. GAPDH served as the internal control for data normalization. **D** Immunohistochemical staining of the distribution of CAMTA1 (top two rows) and ki67 (bottom two rows) in normal and colorectal cancer tissues from two patients. Nuclei were stained with hematoxylin. Red boxes represent magnified views, which are shown to the right, bar = 50 μm. **E** CAMTA1 (top panel) and ki67 (bottom panel) immunohistochemistry staining scores (i.e., number of immuno-positive cells) in normal and colorectal cancer tissues. Data are presented as the mean ± S.E.M., *n* = 3. *P < 0.05; ****P < 0.0001 by one-way ANOVA, followed by Tukey’s HSD test.

To investigate the levels of CAMTA1 and NFATc4, we performed qPCR, western blotting, and immunohistochemical staining experiments using normal and colorectal cancer tissues collected from patients during surgery. The levels of *CAMTA1* mRNA were significantly lower in colorectal cancer tissues than that in paired adjacent normal tissues (Fig. 1A), whereas the *NFATC4* mRNA level was significantly higher in colorectal cancer tissues (Fig. 1B). The results of western blotting (Fig. 1C, please see Supplementary Materials for original western blot data) and immunohistochemical staining (Fig. 1D) experiments, which measured the CAMTA1 level in normal and colorectal cancer tissues from different patients, agreed with those of qPCR, confirming the significant decrease in the CAMTA1 level in human colorectal cancer tissues. Immunohistochemistry staining scores revealed a lower percentage of CAMTA1 immuno-positive cells in colorectal cancer tissues, when compared to that in normal tissues but a higher percentage of Ki-67, a cell proliferation marker, immuno-positive cells [16]. We analyzed the correlation between the expression of CAMTA1 and OS based on the relevant cancer data in the TCGA database through the UALCAN (http://ualcan.path.uab.edu/index.html) website. The results showed that the expression of CAMTA1 in CRC was positively correlated with the Overall Survival probability (Supplementary Fig. 1, *p* = 0.021).

### CAMTA1 knockdown promotes colorectal cancer cell proliferation and invasion in vitro

**Fig. 2:**
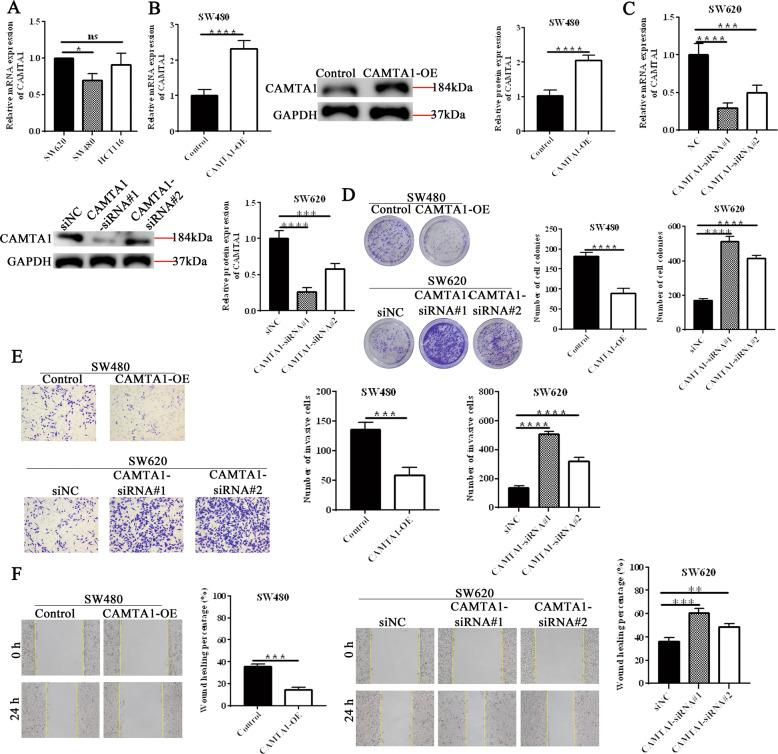
*CAMTA1* knockdown promotes colorectal cancer cell proliferation and invasion in vitro. **A** qPCR analysis of the *CAMTA1* mRNA level in different colorectal cancer cell lines. **B** *CAMTA1* overexpression (OE) efficiency in SW480 cells, as determined by qPCR (left panel) and western blotting (right panel) analyses. Transfection of an empty vector served as the control in overexpression experiments. GAPDH served as the internal control for data normalization. **C** *CAMTA1* knockdown efficiency in SW620 cells, as determined by qPCR (left panel) and western blotting (right panel) analyses. Transfection of a nontargeting siRNA (NC/si-NC) served as the control, and two different siRNAs (siRNA#1, siRNA#2) were used for *CAMTA1* knockdown in RNAi experiments. **D** Colony formation assay results of the effects of *CAMTA1* overexpression and knockdown on SW480 and SW620 cell proliferation, respectively. Graphs (right panels) summarize results from three independently-performed experiments. **E** Migration assay results of the effects of *CAMTA1* overexpression and knockdown on SW480 and SW620 cell invasion, respectively. Graphs (right panels) summarize results from three independently-performed experiments. **F** Wound healing assay results of the effects of *CAMTA1* overexpression and knockdown on SW480 and SW620 cell migration at 24 h, respectively. Yellow lines represent regions where there were no cells. Graphs (right panels) summarize results from three independently-performed experiments. Data are presented as the mean ± S.E.M., *n* = 3. ns not significant; *P < 0.05; **P < 0.01; ***P < 0.001; ****P < 0.0001 by one-way ANOVA, followed by Tukey’s HSD test.

To understand the role of CAMTA1 in colorectal cancer, we conducted *CAMTA1* overexpression and knockdown experiments and examined the effects of these manipulations on cell migration and invasion in vitro. In qPCR experiments, we found that *CAMTA1* was expressed by SW620, SW480, and HCT116 cells at comparable levels (Fig. 2A), and SW480 and SW620 cell lines were selected for further studies as representative colorectal cancer cells. Compared with respective controls, *CAMTA1* mRNA and protein levels were increased in *CAMTA1*-overexpressed SW480 cells (Fig. 2B) and decreased in *CAMTA1*-silenced SW620 cells (Fig. 2C, please see Supplementary Materials for original western blot data), indicative of successful overexpression and knockdown (i.e., for both siRNAs, *Camta1*-siRNA#1 and *Camta1*-siRNA#2) in vitro. The results of colony formation assays revealed that the proliferation of *CAMTA1*-overexpressed SW480 cells (Fig. 2D) was decreased (i.e., resulting in a lower number of colonies compared with the control), whereas that of *CAMTA1*-silenced SW620 cells (Fig. 2D) was increased (i.e., resulting in a higher number of colonies compared with the control). Furthermore, the results of Transwell assays demonstrated that the invasion of *CAMTA1*-overexpressed SW480 was significantly decreased (Fig. 2E) and that of *CAMTA1*-silenced SW620 cells was significantly increased (Fig. 2E) compared with respective controls. These results agreed with those of wound healing assays, which showed that the migration of *CAMTA1*-overexpressed SW480 cells was decreased (Fig. 2F) and that of *CAMTA1*-silenced SW620 cells was increased (Fig. 2F) compared with respective controls. Taken collectively, these results indicate that CAMTA1 can inhibit colorectal cancer cell proliferation, invasion, and migration, which is consistent with its function as a tumor suppressor.

### CAMTA1 knockdown promotes colorectal cancer cells oxaliplatin resistance in vitro

**Fig. 3:**
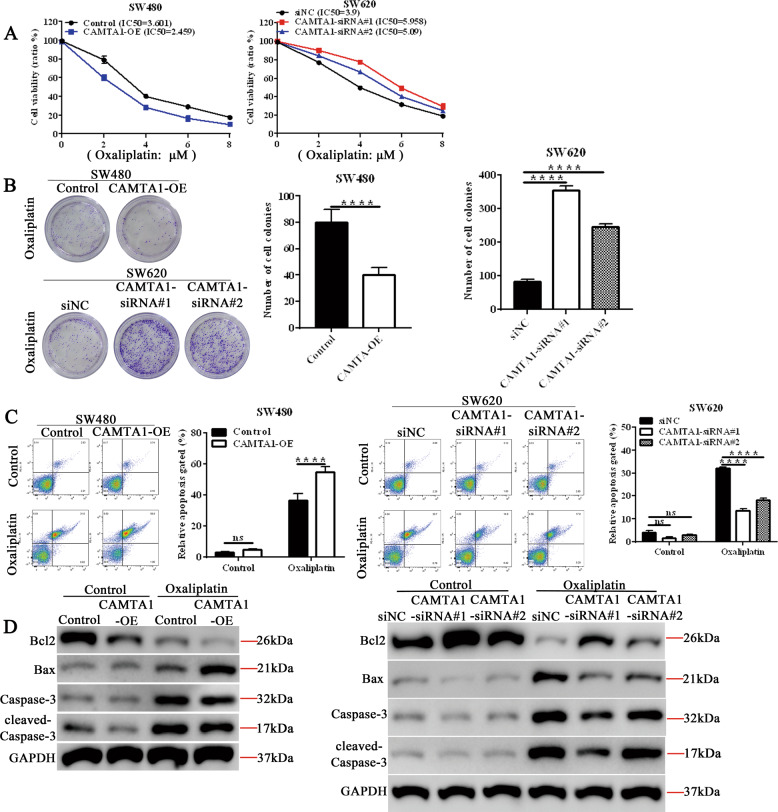
Oxaliplatin promotes apoptosis in *CAMTA1*-overexpressed colorectal cancer cells in vitro. **A** CCK8 assay results of the effects of different concentrations of oxaliplatin on the viability of *CAMTA1*-overexpressed SW480 (left panel) and *CAMTA1*-silenced SW620 (right panel) cells. Transfection of an empty vector served as the control in overexpression (OE) experiments. Transfection of a nontargeting siRNA (si-NC) served as the control, and two different siRNAs (siRNA#1, siRNA#2) were used for *CAMTA1* knockdown in RNAi experiments. **B** Colony formation assay results of the effects of *CAMTA1* overexpression (top panel) and knockdown (bottom panel) on the resistance of SW480 and SW620 cells to 4 μM oxaliplatin, respectively. Graphs (right panels) summarize results from three independently-performed experiments. **C** Flow cytometry results of apoptosis in *CAMTA1*-overexpressed SW480 (top panel) and *CAMTA1*-silenced SW620 (bottom panel) cells treated with 4 μM oxaliplatin. **D** Western blotting analysis of the levels of apoptosis-related proteins in *CAMTA1*-overexpressed SW480 (right panel) and *CAMTA1*-silenced SW620 (left panel) cells treated with 4 μM oxaliplatin. GAPDH served as the internal control for data normalization. Data are presented as the mean ± S.E.M., *n* = 3. **P < 0.01; ****P < 0.0001 by one-way ANOVA, followed by Tukey’s HSD test.

To determine whether *CAMTA1* overexpression or knockdown could affect the survival of oxaliplatin-treated colorectal cancer cells, we assessed the cell viability, apoptosis, colony formation, and performed western blotting experiments in which the levels of apoptosis-related proteins were measured. The viability of SW480 and SW620 cells (i.e., controls/transfected with empty vector or nontargeting siRNA, *CAMTA1*-overexpressed, *CAMTA1*-silenced) treated with increasing concentrations of oxaliplatin (2–8 μM) for 24 h was significantly affected (Fig. 3A). The results of colony formation assay showed that after treatment with 4 μM oxaliplatin, the proliferation of CAMTA1-overexpressed SW480 cells was significantly lower than that of the respective control group, while the proliferation of CAMTA1-silenced SW620 cells was significantly higher than that of the respective control group (Fig. 3B). In flow cytometry experiments, we found that the apoptotic rate was higher in CAMTA1-overexpressed SW480 cells than that in the control, whereas it was lower in CAMTA1-silenced SW620 cells after the treatment of oxaliplatin (Fig. 3C). These results agreed with those of western blotting experiments. After oxaliplatin treatment, the expression of Bcl2 in SW480 cells overexpressing CAMTA1 was significantly lower than that of the control group, and the levels of Bax, caspase-3, and cleaved-caspase-3 were significantly higher, respectively, In SW620 cells, compared with the corresponding control group, Bcl2 levels in CAMTA1-silenced cells increased, but Bax, caspase-3, and cleaved-caspase-3 levels decreased (Fig. 3D, please see Supplementary Materials for original western blot data).

### CAMTA1 knockdown upregulates NFATc4 expression in colorectal cancer cell in vitro

**Fig. 4:**
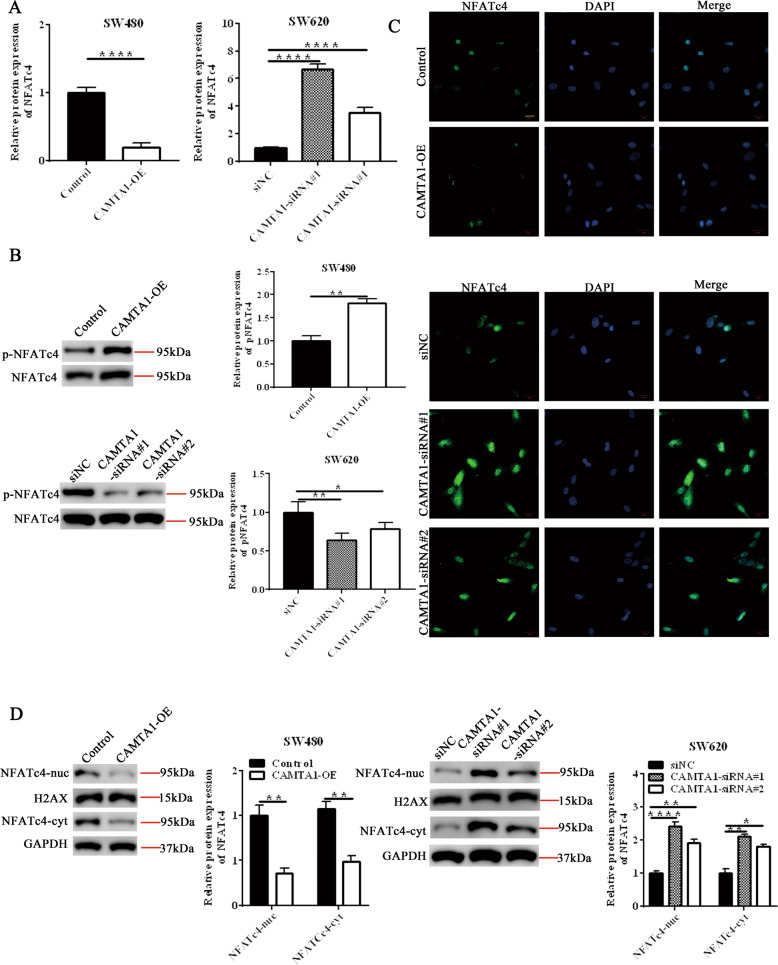
*CAMTA1* knockdown upregulates *NFATc4* expression in colorectal cancer cells in vitro. **A** qPCR analysis of the *NFATc4* mRNA level after *CAMTA1* overexpression (left panel) and knockdown (right panel) in SW480 and SW620 cells, respectively. Transfection of an empty vector served as the control in overexpression (OE) experiments. Transfection of a nontargeting siRNA (si-NC) served as the control, and two different siRNAs (siRNA#1, siRNA#2) were used for *CAMTA1* knockdown in RNAi experiments. **B** Western blotting analysis of p-NFATc4 relative to the total of the NFATc4 protein after *CAMTA1* overexpression (top panel) or knockdown (bottom panel) in SW480 and SW620 cells, respectively. Graphs (right panel) summarize results from three independently-performed experiments. **C** Immunofluorescent staining of the distribution of NFATc4 (green fluorescence) in *CAMTA1*-overexpressed SW480 (top panel) and *CAMTA1*-silenced SW620 (bottom panel) cells. Nuclei (blue fluorescence) were stained with DAPI. Representative images were merged, bar = 20 μm. **D** Western blotting analysis of the NFATc4 level in nuclear (nuc) and cytosolic (cyt) fractions from *CAMTA1*-overexpressed SW480 (top panel) and *CAMTA1*-silenced SW620 (bottom panel) cells. Graphs (right and bottom panels) summarize results from three independently-performed experiments. Data are presented as the mean ± S.E.M., *n* = 3. ns not significant; *P < 0.05; **P < 0.01; ****P < 0.0001 by one-way ANOVA, followed by Tukey’s HSD test.

In order to further explore the mechanism of CAMTA1 regulating oxaliplatin resistance in colorectal cancer, we predicted the downstream target genes of CAMTA1 and analyzed the expression of NFATc4. We performed western blotting and immunofluorescent staining experiments using CAMTA1-overexpressed and CAMTA1-silenced colorectal cancer cells. The NFATc4 level in CAMTA1-overexpressed SW480 cells was significantly lower than that in the control, whereas the NFATc4 level in CAMTA1-silenced SW620 cells was significantly higher (Fig. 4A). These results indicated that NFATc4 was negatively regulated by CAMATA1. We performed western blot experiments to further confirm the regulation of NFATc4 phosphorylation by CAMTA1. The protein level of pNFATc4 increased in CAMTA1-overexpressed SW480 cells and the protein level of pNFATc4 decreased in CAMTA1-silenced SW620 cells. The results indicate that CAMTA1 positively regulates the phosphorylation of NFATc4. (Fig. 4B, please see Supplementary Materials for original western blot data). To confirm the changes in NFATc4 expression and to expand our understanding of NFATc4 function in CAMTA1-overexpressed or -silenced colorectal cancer cells, we performed immunofluorescent staining experiment to detect the expression of NFATc4 in the cytoplasm and nucleus after overexpression or silence of CAMTA1. We found that the levels of NFATc4-nuc and NFATc4-cyt in SW480 cells overexpressing CAMTA1 were lower than those in the corresponding control; while the levels of NFATc4-cyt and NFATc4-nuc in SW620 cells where CAMTA1 was silenced were higher (Fig. 4C). We prepared nuclear and cytosolic fractions and examined the NFATc4 level. The result agreed with that of immunofluorescent staining experiment in which the NFATc4 level was significantly decreased and increased in CAMTA1-overexpressed SW480 and CAMTA1-silenced SW620 cells, respectively (Fig. 4D, please see Supplementary Materials for original western blot data).

### NFATc4 mediates oxaliplatin resistance in colorectal cancer cells regulated by CAMTA1 in vitro

**Fig. 5:**
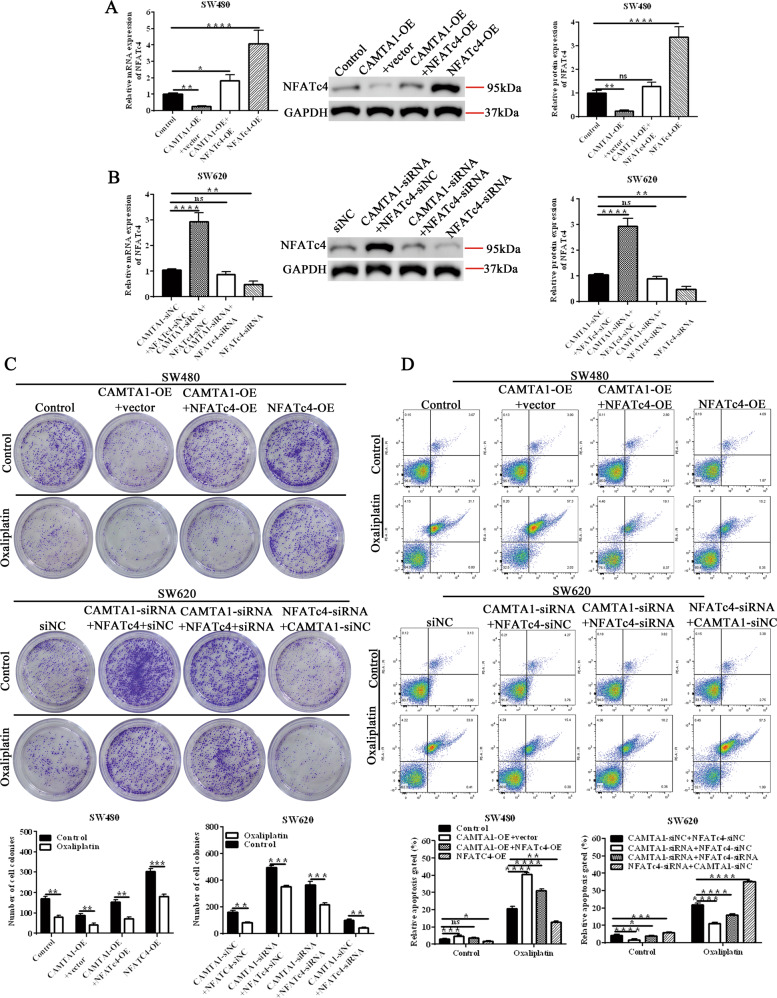
NFATc4 mediates oxaliplatin resistance in *CAMTA1*-silenced colorectal cancer cells in vitro. **A**, **B** qPCR and western blotting analysis of the *NFATc4* mRNA level in mock-overexpressed, *CAMTA1* + vector overexpressed, *CAMTA1* + *NFATc4*-overexpressed, and *NFATc4*-overexpressed SW480 cells (top panel), and qPCR analysis of the *NFATc4* mRNA in *CAMTA1*-si-NC + *NAFTc4*-si-NC-silenced, *CAMTA1* + *NFATc4*-si-NC-silenced, *CAMTA1*-si-NC + *NFATc4*-silenced, and *CAMTA*1 + *NFATc4*-silenced SW620 cells (bottom panel). Transfection of an empty vector (vector) served as the control in overexpression experiments, whereas transfection of a nontargeting siRNA (si-NC) served as the control in RNAi experiments. GAPDH served as the internal control for data normalization. **C** Colony formation assay results of the effects of mock-overexpression, *CAMTA1* + vector overexpression, *CAMTA1* + *NFATc4* overexpression, and *NFATc4* overexpression (top panel), and the effects of *CAMTA1*-si-NC + *NAFTc4*-si-NC-kncokdown, *CAMTA1* + *NFATc4*-si-NC-knockdown, *CAMTA1*-si-NC + *NFATc4*-knockdown, and *CAMTA*1 + *NFATc4*-knockdown (bottom panel) on the resistance of SW480 and SW620 cells to 4 μM oxaliplatin, respectively. **D** Flow cytometry results of apoptosis in mock-overexpressed, *CAMTA1* + vector overexpressed, *CAMTA1* + *NFATc4* overexpressed, and *NFATc4* overexpressed SW480 cells (top panel), and apoptosis in *CAMTA1*-si-NC + *NAFTc4*-si-NC-silenced, *CAMTA1* + *NFATc4*-si-NC-silenced, *CAMTA1*-si-NC + *NFATc4*-silenced, and *CAMTA*1 + *NFATc4*-silenced SW620 cells (bottom panel) treated with 4 μM oxaliplatin. Data are presented as the mean ± S.E.M., *n* = 3. ns not significant; *P < 0.05; **P < 0.01; ***P < 0.001; ****P < 0.0001 by one-way ANOVA, followed by Tukey’s HSD test.

To further clarify the regulation of CAMTA1 on NFATc4 and its role in oxaliplatin resistance in colorectal cancer, CAMTA1 and NFATc4 were overexpressed or interfered in SW480 cells and SW620 cells, respectively. It was found by qPCR and western blot that CAMTA1 negatively regulates the expression of NFATc4 (Fig. 5A, B, please see Supplementary Materials for original western blot data). In order to clarify the role of NFATc4 in the oxaliplatin resistance of colorectal cancer regulated by CAMTA1, we conducted clone formation experiments and apoptosis detection respectively. Clone formation experiments showed that in SW480 cells, overexpression of NFATc4 increased the proliferation ability of cells, while overexpression of CAMTA1 weakened this ability. In addition, overexpression of NFATc4 also enhanced the resistance of cells to oxaliplatin. Similarly, overexpression of CAMTA1 weakened this ability. In SW620 cells, knockdown of NFATc4 reduced the proliferation ability of cells, knockdown of CAMTA1 rescued the decrease of cell proliferation caused by knockdown of NFATc4, and knockdown of CAMTA1 also rescued cells caused by knockdown of NFATc4 decreased resistance to oxaliplatin (Fig. 5C). Flow cytometry detection of cell apoptosis found that after oxaliplatin treatment of SW480 cells, the overexpression of NFATc4 reduced cell apoptosis, while the overexpression of CAMTA1 induced apoptosis of NFATc4 overexpressing cells. In SW620 cells, knockdown of NFATc4 increased apoptosis, and knockdown of CAMTA1 rescued this change (Fig. 5D). In conclusion, the negative regulation of NFATc4 by CAMTA1 is involved in the oxaliplatin resistance of colorectal cancer.

### CAMTA1 directly interacts with NFATc4 and PPP3CA and upregulates NFATc4 expression in colorectal cancer cells in vitro

**Fig. 6:**
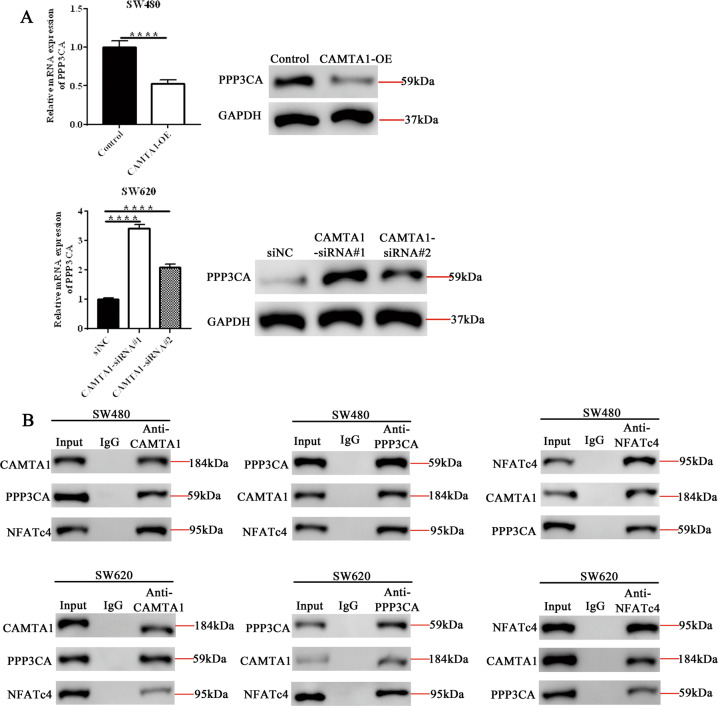
CAMTA1 directly interacts with NFATC4 and PPP3CA and upregulates *PPP3CA* expression in colorectal cancer cells in vitro. **A** qPCR (left panel) and western blotting (right panel) analyses of PPP3CA mRNA and protein levels in *CAMTA1*-overexpressed SW480 (top panel) and *CAMTA1*-silenced cells (bottom panel), respectively. GAPDH served as the internal control for data normalization. **B** Co-immunoprecipitation and western blotting analyses of interactions among CAMTA1, NFATC4, and PPP3CA in SW480 (top panel) and SW620 (bottom panel) cells. Input IgG served as the internal control.

PPP3CA is one of the genes encoding calmodulin-binding subunit calcineurin A, which regulates the Cn/NFAT signaling pathway. PPP3CA regulates the phosphorylation of NFATc4. In order to further clarify the mechanism of action of CAMTA1/NFATc4 in oxaliplatin resistance in colorectal cancer, we analyzed the interaction between PPP3CA, CAMTA1, and NFATc4. To determine whether CAMTA1, NFATc4, and PPP3CA interact in colorectal cancer cells, we performed qPCR, western blotting, and co-immunoprecipitation experiments. The *PPP3CA* mRNA and protein levels were decreased and increased in *CAMTA1*-overexpressed SW480 and *CAMTA1*-silenced SW620 cells, respectively (Fig. 6A, please see Supplementary Materials for original western blot data). In co-immunoprecipitation and western blotting experiments, we found that CAMTA1, NFATc4, and PPP3CA interacted, forming a multi-protein complex, in SW480 and SW620 cells (Fig. 6B and Supplementary Fig. 2, please see Supplementary Materials for original western blot data).

### CAMTA1 knockdown promotes colorectal cancer growth and oxaliplatin resistance in nude mice

**Fig. 7:**
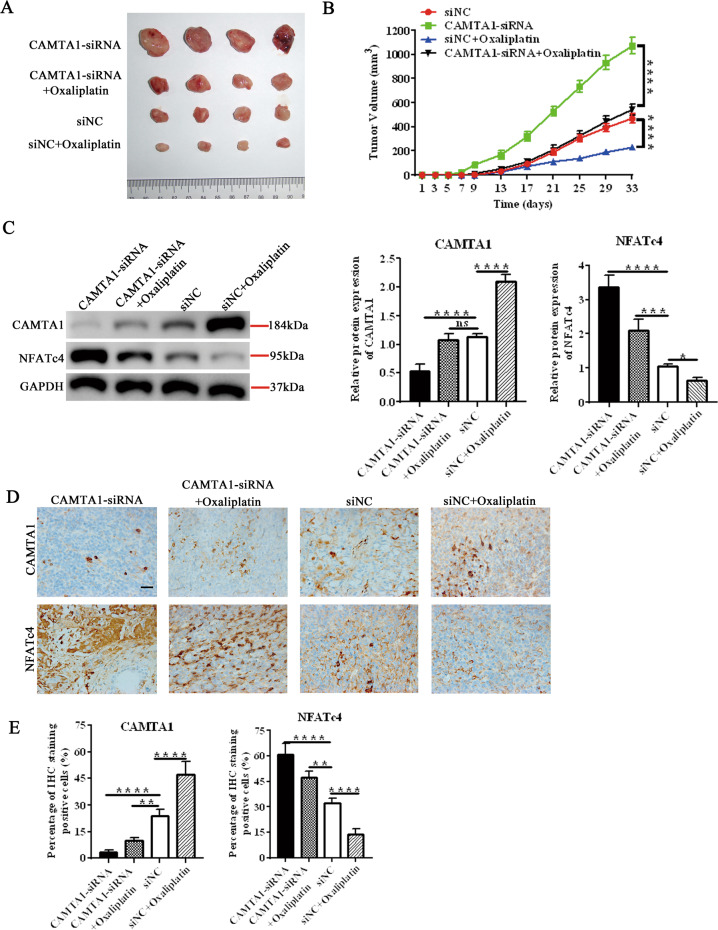
*CAMTA1* knockdown promotes colorectal cancer growth and oxaliplatin resistance in nude mice. **A** Colorectal tumors from representative *Camta1*-silenced mice with and without treatment with oxaliplatin (5 mg/kg body weight). Transfection of a nontargeting siRNA (si-NC) served as the control in RNAi experiments. **B** Colorectal tumor volumes in *Camta1*-silenced mice with and without treatment with oxaliplatin, as indicated above. **C** Western blotting analysis of CAMTA1 and NFATc4 protein levels in *Camta1*-silenced mice with and without treatment with oxaliplatin. GAPDH served as the internal control for data normalization. Graphs (right panel) summarize results from three independently-performed experiments. **D** Immunohistochemical staining of the distribution of CAMTA1 (top row) and NFATc4 (bottom row) in *CAMTA1*-silenced mice with and without treatment with oxaliplatin. Nuclei were stained with hematoxylin, bar = 20 μm. **E** CAMTA1 (left panel) and NFATc4 (right panel) immunohistochemical staining scores (i.e., number of immuno-positive cells) in *Camta1*-silenced mice with and without treatment with oxaliplatin. Data are presented as the mean ± S.E.M., *n* = 3. ns not significant; *P < 0.05; **P < 0.01; ***P < 0.001; ****P < 0.0001 by one-way ANOVA, followed by Tukey’s HSD test.

To understand these in vitro findings in an in vivo context, as well as to confirm the role of CAMTA1 in colorectal cancer growth and oxaliplatin resistance, we established a xenograft mouse model. Compared with the control (i.e., siNC), tumor volume was increased in mice in which Camta1 expression was silenced. After treatment with oxaliplatin, the tumor volume of mice with silenced CAMTA1 expression was significantly higher than that of the control group (Fig. 7A, B). In western blotting experiments, we found that the CAMTA1 protein level in the cancer tissue of the mice treated with oxaliplatin was higher than that in the untreated group. By contrast, NAFTc4 protein levels were lower in cancer tissues of the mice treated with oxaliplatin than that in the untreated group (Fig. 7C, please see Supplementary Materials for original western blot data). These results agreed with those of immunohistochemical staining experiments, which showed similar patterns of Camta1 and Nfatc4 expression (Fig. 7D). Compared with the control, the highest percentage of CAMTA1 immuno-positive cells was found in colorectal cancer tissues from mice treated with oxaliplatin, whereas the lowest percentage was found in colorectal cancer tissues in which Camta1 expression was silenced (Fig. 7E). By contrast, compared with the control, the highest percentage of NFATc4 immuno-positive cells was found in colorectal cancer tissues from mice in which Camta1 expression was silenced, whereas the lowest percentage was found in colorectal cancer tissues from mice treated with oxaliplatin (Fig. 7E).

## Discussion

It is not surprising that the incidence and prevalence of colorectal cancer are gradually increasing worldwide. More concerning is the fact that colorectal cancer is often diagnosed at late stages, as most individuals do not have symptoms until the disease has progressed and possibly metastasized. For this reason, regular screenings are important, and further studies are warranted to identify and develop new treatment modalities with the goal of improving the prognosis. Previously, studies have reported that CAMTA1, a transcription factor, plays a key role in a variety of cancers, although its mechanism of action in colorectal cancer is not known. Here, we found that *CAMTA1* mRNA and protein levels in colorectal cancer tissues from different patients were significantly lower than those in paired adjacent normal tissues. By contrast, the *NFATC4* mRNA level in colorectal cancer tissues from different patients was significantly higher than that in paired adjacent normal tissues. The mRNA level of NFATc4is negatively correlated with the mRNA level of CAMTA1 in colorectal cancer tissue. These findings clearly indicate that the low expression of *CAMTA1*, a tumor suppressor, may promote tumor growth and development, which is further supported by the high percentage of KI-67 immuno-positive cells in colorectal cancer compared with normal tissues. KI-67 is a marker of cell proliferation, and a high KI-67 score associates with advanced tumorigenesis.

To understand the role of CAMTA1 in colorectal cancer, we conducted *CAMTA1* overexpression and knockdown experiments and examined the effects of these manipulations on cell migration and invasion using representative colorectal cancer cell lines. Compared with respective controls, we found that the invasive and migratory ability of *CAMTA1*-overexpressed SW480 cells was significantly decreased, whereas that of *CAMTA1*-silenced SW620 cells was significantly increased, confirming previous findings that indicated CAMTA1 to function as a tumor suppressor. Thereafter, the effects of *CAMTA1* overexpression and knockdown on the resistance of colorectal cancer cells to oxaliplatin, a common chemotherapeutic drug, were examined. We hypothesized that overexpression of *CAMTA1* would render colorectal cancer cells more sensitive to oxaliplatin (i.e., more sensitive to apoptosis) than overexpression of an empty vector, and this would be clearly supported by the finding that the ability of *CAMTA1*-overexpressed cells to inhibit tumor growth would be significantly greater than that of control cells. Indeed, we found that the apoptotic rate was higher in *CAMTA1*-overexpressed SW480 cells treated with oxaliplatin than that in the control, whereas the apoptotic rate was lower in *CAMTA1*-silenced SW620 cells treated with oxaliplatin. These results agreed with those of western blotting experiments in which the levels of Bcl2, a regulator of apoptosis, and caspase-3, an executioner of apoptosis, were decreased and increased, respectively. Taken collectively, these findings indicate that CAMTA1 can inhibit tumor growth and development, and that CAMTA1 is involved in the resistance of colorectal cancer to oxaliplatin.

To understand the role of NFATc4, a transcription factor, in colorectal cancer, as well as to determine whether its expression could be affected by the manipulation of *CAMTA1* expression, we used *CAMTA1*-overexpressed and *CAMTA1*-silenced colorectal cancer cells. The NFATc4 level was significantly downregulated in *CAMTA1*-overexpressed SW480 cells, when compared to the control, whereas the NAFTc4 level in *CAMTA1*-silenced SW620 cells was significantly higher. Interestingly, these results were reversed in the case of pNFATc4 whose level was increased and decreased in *CAMTA1*-overexpressed SW480 cells and *CAMTA1*-silenced SW620 cells, respectively. In addition, we found that the levels of NFATc4-nuc and NFATc4-cyt were lower in *CAMTA1*-overexpressed SW480 cells than those in respective controls, whereas the NFATc4-nuc level and the NFATc4-cyt level were higher in *CAMTA1*-silenced SW620 cells compared with respective controls. A previous study has reported that phosphorylated NFATc4, which is inactive, is confined to the cytosol [17]. As such, a re-evaluation of the results from western blotting experiments would reveal that NAFc4 is mostly dephosphorylated, and thus, activated in the nuclei of *CAMTA1*-silenced SW620 cells. Further studies are necessary to elucidate the roles of NFATc4 phosphorylation and dephosphorylation in colorectal cancer.

To further clarify the regulation of CAMTA1 on NFATc4 and its role in oxaliplatin resistance in colorectal cancer, CAMTA1 and NFATc4 were overexpressed or interfered with SW480 cells and SW620 cells, respectively. Compared with respective controls, Nfatc4 mRNA and protein levels were decreased in Camta1-overexpressed SW480 cells and increased in SW620 cells silenced by CAMTA1. In colorectal cancer cells, CAMTA1 negatively regulates the expression of NFATc4. To assess the effect of NFATc4 in oxaliplatin resistance in colorectal cancer, we constructed CAMTA1 overexpression, NFATc4 overexpression, and CAMTA1 + NFATc4 overexpression cells in SW620 cells, CAMTA1 knockdown, NFATc4 knockdown, and CAMTA1 + NFATc4 knockdown cells in sw480 cells (i.e., for SW480 cells, CAMTA1-OE, CAMTA1-OE + NFATc4-OE, NFATc4-OE; for SW620 cells, CAMTA1-siRNA, CAMTA1-siRNA+NFATc4-siRNA, NFATc4-siRNA). We performed clone formation and apoptosis assays in these cells. In the presence of oxaliplatin, overexpression of NFATc4 increased proliferation, colony formation, and decreased apoptosis. However, overexpression of CAMTA1 reversed this effect and rescued the cells from oxaliplatin resistance (Fig. 5). We also confirmed such observations in NFATc4 knockdown cells, indicating that the negative regulation of NFATc4 by CAMTA1 is related to oxaliplatin resistance in colorectal cancer.

To further confirm CAMTA1’s role in colorectal cancer, we established xenograft models and silenced CAMTA1. In these silenced mice, oxaliplatin treatment had no effect and displayed significantly higher tumor volume, indicating oxaliplatin resistance (Fig. 7). Further, silencing of CAMPTA1 increased the NAFTc4 levels in the colorectal cancer tissues of mice. Taken collectively, these findings indicate that CAMTA1 overexpression can render colorectal cancer cells more sensitive to oxaliplatin, and thus, this approach may hold clinical promise in the future, although prolonged gene overexpression in rapidly growing tumors is presently a challenge. Further functional studies are required to understand the roles of CMATA1 and NFATc4, as well as PPP3CA, in colorectal cancer.

## Materials and methods

### Ethics statement

Human colorectal cancer specimens were obtained after all patients provided with written informed consents. No patient received neoadjuvant therapy. This study has an approval from the Institutional Review Committee of the Ruijin Hospital of Shanghai Jiaotong University of China and performed in accordance with the tenets of the Declaration of Helsinki.

### Patient specimens

Tumor and paired adjacent normal tissues from colon cancer patients treated at the Ruijin Hospital of Shanghai Jiaotong University of China were collected. For quantitative polymerase chain reaction (qPCR) analysis, specimens were minced and stored in RNA*later* (ThermoFisher Scientific, Waltham, MA, USA) for the isolation of total RNA. For western blotting and immunohistochemistry analyses, protein was isolated from specimens by freezing them in liquid nitrogen and fixed in 4% (w/v) paraformaldehyde for paraffin embedding and sectioning, respectively.

### Cell culture

Colon cancer cell lines (SW620, SW480, and HCT116) were obtained from ATCC (American Type Culture Collection, Manassas, VA, USA) and cultured in RPMI 1640 medium (Sigma, St. Louis, MO, USA) containing 10% (v/v) fetal bovine serum (GIBCO, Grand Island, NY, USA), 1% (v/v) glutamine (GIBCO), and 1% (v/v) penicillin/streptomycin (GIBCO) at 37 °C humidified incubator with an atmosphere of 5% (v/v) CO_2_. Thereafter, cells were used for further studies, as indicated below.

### Cell viability assay

The Cell Counting-8 kit (Dojindo Molecular Technologies, Rockville, MD, USA) was used to assess the cell viability, by following the manufacturer’s protocol. In brief, cells (1 × 10^4^ cells/ml) were resuspended in RPMI 1640 medium containing 10% (v/v) FBS, 1% (v/v) glutamine, and 1% (v/v) penicillin/streptomycin, and 200-μl aliquots of the cell suspension were seeded in 96-well plates and incubated overnight, as indicated. Thereafter, cells were treated with different concentrations of oxaliplatin (2, 4, 6, 8 μmol/l) for 5 days. Control cells were not treated with the drug. The oxaliplatin-supplemented media was changed daily. Determination of cell viability was performed daily by assessing the absorbance readings at 450 nm with an Epoch microplate spectrophotometer (BioTek, Winooski, VT, USA). Further, the half-maximal inhibitory concentration (IC_50_) was assessed with data obtained on day 5 and GraphPad Prism 7 software (La Jolla, CA, USA).

### Colony formation assay

The colony formation assay was performed using a previously published protocol [18]. Briefly, resuspended cells in RPMI 1640 medium containing 10% (v/v) FBS, 1% (v/v) glutamine, and 1% (v/v) penicillin/streptomycin were seeded in 12-well plates and cultured for 12 d, as indicated. Thereafter, cells were treated with oxaliplatin (4 μmol/l) for 24 h, and prechilled methanol was used to fix the colonies which were further stained with 0.5% (w/v) crystal violet. For each well, five random fields were viewed under an inverted light microscope (Zeiss, Oberkochen, Germany) and used to determine the number of colonies, which was compared with respective controls. Control cells were not treated with the drug.

### Transwell assays

The cell migration assay was performed using a protocol that was previously published [19]. In brief, cells (4 × 10^4^ cells) were added to the upper chambers of Corning Costar 8-μm-pore size Transwell units (Cambridge, MA, USA) and cultured for 24 h, as indicated. Cells were treated with oxaliplatin (4 μmol/l) for 24 h. Further, 600 μl of the RPMI 1640 medium containing 10% (v/v) FBS, 1% (v/v) glutamine, and 1% (v/v) penicillin/streptomycin were added to the lower chambers. Migration of cells through the membranes was allowed for 20 h at 37 °C.

The cell invasion assay was performed using a previously published protocol [20]. In brief, cells (4 × 10^4^ cells) were added to the upper chambers of Corning Costar Matrigel-coated 8-μm-pore size Transwell units and cultured for 24 h, as indicated. Cells were treated with oxaliplatin (4 μmol/l) for 24 h. A total 600 μl of RPMI 1640 medium containing 10% (v/v) FBS, 1% (v/v) glutamine, and 1% (v/v) penicillin/streptomycin were added to the lower chambers. Thereafter, prechilled methanol was used to fix the cells on the underside of membranes which were further stained with 0.5% (w/v) crystal violet. For each Transwell unit, five random fields were viewed using an inverted light microscope (Nikon, Tokyo, Japan) and used to determine the percentages of migratory and invasive cells, which were compared with respective controls. Control cells were not treated with the drug.

### Wound healing assay

Using a previously published protocol wound healing assay was performed [18]. In brief, cells were resuspended in RPMI 1640 medium containing 10% (v/v) FBS, 1% (v/v) glutamine, and 1% (v/v) penicillin/streptomycin, seeded in 6-well plates, and cultured until 70–80% confluent. Cells were treated with oxaliplatin (4 μmol/l) for 24 h. Thereafter, each well was scratched with a 100-μl pipette tip to create a cell-free area, cells were rinsed with medium, and plates were incubated for 24 h. For each well, five random fields were viewed under an inverted light microscope and used to determine the percentage of migratory cells, which was compared with respective controls. Control cells were not treated with the drug.

### Apoptosis assay

For the apoptosis assay, the annexin V-fluorescein isothiocyanate (FITC)/propidium iodide (PI) kit (Abcam, Cambridge, MA, USA) was used. In brief, cells were treated with oxaliplatin (4 μmol/l) for 24 h, detached with accutase (Sigma), and washed twice with prechilled phosphate-buffered saline (PBS, 137 mM NaCl, 2.7 mM KCl, 10 mM Na_2_HPO_4_, 1.8 mM KH_2_PO_4_, pH 7.5). Thereafter, cells (1 × 10^6^ cells/ml) were resuspended in annexin V binding buffer, and 100-μl aliquots of the cell suspension were combined with 5 μl of annexin V-FITC and 5 μl of PI based on the instructions from the manufacturer. Cells were then analyzed by flow cytometry (Beckman Coulter, Pasadena, CA, USA). The percentages of apoptotic cells, early apoptotic cells (Annexin V+/PI−), and late apoptotic/necrotic cells (Annexin V+/PI+) were determined and compared with respective controls. Control cells were not treated with the drug.

### Immunohistochemical staining

Initially, we deparaffinized 3-micron-thick sections, rehydrated, and endogenous peroxidase activity was blocked via incubation with 3% hydrogen peroxide for 15 min at room temperature. Antigen retrieval was performed using sodium citrate buffer (pH 6.0) for 3 min in a pressure cooker. Blocking of sections was performed using 5% bovine serum albumin (BSA) for 30 mins at room temperature to remove non-specific binding. Sections were further incubated with primary antibodies for CAMTA1 (1:200; Bioss Antibodies, Woburn, MA, USA; bs-5995R) and KI-67 overnight at 4 °C. Thereafter, secondary antibody incubations of sections were carried out for 1 h at 37 °C. Finally, a chromogenic reaction was developed with DAB, and counterstaining of the sections was performed with hematoxylin. Control tissue sections were incubated with non-immune rabbit IgG under identical conditions.

### Immunofluorescent staining

Fixation of the cells was performed with 4% (v/v) paraformaldehyde followed by permeabilization with 0.1% (v/v) Triton X-100. Further, blocking of the cells was performed with 2% (v/v) bovine serum albumin. The cells were incubated with an anti-NFATC4 primary antibody (1:100; Santa Cruz Biotechnology, Santa Cruz, CA, USA; sc-271597) in PBS containing 0.5% (w/v) BSA overnight. Coverslips were washed three times (15 min in total) with PBS. Incubation with Alexa Fluor 488 secondary antibody was performed (1:500; Abcam, Cambridge, MA, USA; ab150077), washed with PBS, mounted with medium containing 4′6′-diamidino-2-phenylindole (DAPI, Vector Labs, Burlingame, CA, USA), and viewed under a fluorescent microscope (Olympus, Shinjuku, Japan). Control cells were incubated with non-immune rabbit IgG under identical conditions.

### Quantitative polymerase chain reaction (qPCR) analysis

Using previously described protocol, qPCR was performed [21]. TRIzol reagent was used to isolate the total RNA (Invitrogen/Life Technologies, Carlsbad, CA, USA) based on the manufacturer’s instructions. Thereafter, 2 μg of RNA was reverse transcribed using the PrimeScript RT kit (Takara, Dalian, China). SYBR Premix Ex *Taq* II (Tli RNaseH Plus, Takara) and gene-specific primers were used for qPCR, which was performed with the LightCycler 96 system (Roche). The following primers were used: *Camta1* (forward, 5′-GAAGAGCGTTTCCCAAAGTGT-3′; reverse, 5′-CATTAGTGTTCCAGCGGTGC-3′; GenBank accession no. NM_001349625.2), *Ppp3ca* (forward, 5′-TTGCTGGATATTGATGCGCC-3′; reverse, 5′-GCCCACAAATACAGCACACA-3′; GenBank accession no. NM_001130692.2), *Nfatc4* (forward, 5′-AAGGTCGTCTCAGTACAGGC-3′; reverse, 5′-TTGGAGTCTGGCAGGAAGTT-3′; GenBank accession no. NM_001198967.3), and *Gapdh* (forward, 5′-GGGGAGCCAAAAGGGTCATCATCT-3′; reverse, 5′-GACGCCTGCTTCACCACCTTCTTG-3′, GenBank accession no. NM_001256799.3). Melting curve analysis and agarose gel electrophoresis of the amplified target was performed to assess the specificity of each. The relative mRNA level was determined using the comparative Ct method [22]. *Gapdh* served as the internal control for the normalization of target gene levels.

### Small interfering (si) RNA transfection and target gene silencing

Cells (2 × 10^5^ cells) were resuspended in RPMI 1640 medium containing 10% (v/v) FBS, 1% (v/v) glutamine, and 1% (v/v) penicillin/streptomycin, seeded in 12-well plates, and cultured for 24 h, as indicated. To ensure target gene knockdown specificity, siRNAs were titrated, and the lowest effective concentrations were used for further experiments. Cells were transfected with mouse *Camta1* siRNA (5 pmol; siRNA#1 5′-AGCAAATTCCGAAGTTACTATGA-3′, siRNA#2 5′-TTCCGAAGTTACTATGAACAAAA-3′; ThermoFisher Scientific) or mouse *Nfatc4* siRNA (5 pmol; 5′-CGCTGCTAATTGGGTTCATGTGT-3′; ThermoFisher Scientific) for 24 h. siRNAs were diluted in Opti-MEM I reduced-serum medium (ThermoFisher Scientific), and Using the manufacturer’s instructions, we performed transfection of the cells using Lipofectamine 2000 reagent (Invitrogen/Life Technologies). Cell viability was not affected under these conditions. A nontargeting siRNA (si-NC, ThermoFisher Scientific) served as the negative control. Thereafter, cells were harvested and processed for qPCR and western blotting experiments.

### Preparation of overexpression vectors and target gene overexpression

cDNAs encoding mouse *Camta1* (GenBank accession no. NM_001349625.2) and *Nfatc4* (GenBank accession no. NM_001198967.3) were prepared by PCR, sequenced, and separately cloned into the pcDNA3.1 lentiviral expression vector (ThermoFisher Scientific). Lentiviral production was performed as previously described [23]. In brief, lentiviral particles were produced by transiently transfecting the transducing vector, along with two packaging vectors, into 293T cells. Post 48 h of transfection, supernatants were collected and concentrated. For infection, cells were seeded in 12-well plates, cultured until 70–80% confluent, and infected with lentiviral particles and polybrene. GFP-lentiviral particles served as the control. Cells were harvested 48 h after infection and processed for qPCR and western blotting experiments, as well as colony formation, Transwell, and wound healing assays.

### Isolation of nuclear and cytoplasmic fractions

The Minute Cytosolic and Nuclear Extraction kit (Invent Biotechnologies, Inc., Plymouth, MN, USA) was used to obtain nuclear and cytosolic fractions according to the manufacturer’s instructions. BCA Protein Assay kit (Beyotime Institute of Biotechnology, Shanghai, China) was used to assess the concentrations of nuclear and cytosolic proteins. Nuclear and cytoplasmic NFATc4 expression was examined by western blotting. Histone H2A.X served as the internal control for the nuclear fraction.

### Western blotting analysis

Western blotting analysis was performed as previously described 0. The primary antibodies used were as follows: anti-CAMTA1 (1:1000; Bioss Antibodies, Woburn, MA, USA; bs-5995R), anti-NFATc4 (1:500; Santa Cruz Biotechnology; sc-271597), anti-pNFATc4 (1:1000; Santa Cruz Biotechnology; sc-135771), anti-PPP3CA (1:1000; Santa Cruz Biotechnology; sc-17808), anti-histone H2A.X (1:1000; Abcam; ab10475), and anti-glyceraldehyde 3-phosphate dehydrogenase (GAPDH; 1:1000; Santa Cruz Biotechnology; sc-47724). Relative target protein levels were quantified using ImageJ software (National Institutes of Health, Bethesda, MD, USA). GAPDH served as the internal control for the normalization of target protein levels.

### Co-immunoprecipitation assay

For the co-immunoprecipitation assay, radioimmunoprecipitation assay buffer containing broad-spectrum protease inhibitors (50 mM Tris–HCl, 150 mM NaCl, 1% (v/v) Nonidet-40, 0.5% (v/v) sodium deoxycholate, 1 mM EDTA, 0.1% (w/v) sodium dodecyl sulfate (SDS), pH 7.4) was used to lyse the cells. Thereafter, 1 mg of protein was incubated with 3 µg of anti-CAMTA1 IgG, anti-NFATc4 IgG, or anti-PPP3CA IgG overnight at 4 °C on a rotator. Protein A agarose beads (Santa Cruz Biotechnology) were added according to the manufacturer’s instructions, followed by incubation for 2 h at 4 °C. Agarose beads were washed. Immunoprecipitated proteins were extracted with SDS sample buffer (0.125 M Tris, 1% (w/v) SDS, 1.6% (v/v) β-mercaptoethanol, 20% (v/v) glycerol, pH 6.8) and used for western blotting experiments.

### Xenografting of nude mice

*Camta1* expression was silenced in SW620 cells by lentiviral vector-mediated siRNA knockdown. Control cells were transfected with the empty lentiviral vector. Thereafter, we subcutaneously injected the transfected or mock-transfected cells (2 × 10^6^) into the flanks of 6-week-old male BALB/cA-nu mice (Shanghai SLAC Laboratory Animal Co., Ltd., Shanghai, China). After 7 d, mice received normal saline or oxaliplatin (5 mg/kg body weight) subcutaneously once every 3 d. The tumor volume was determined every 3 d using the formula: volume = length × width^2^/2. After 33 d, all mice were sacrificed. Tumors were harvested, weighed, imaged, and used for western blotting and immunohistochemical staining experiments.

### Statistical analysis

Statistical analysis was performed using SPSS Statistics 18.0 software (IBM, Armonk, NY, USA). One-way ANOVA, followed by Tukey’s honestly significant difference (HSD) test, was used for statistical analysis. All experiments were carried out at least three independent times, with similar results obtained each time. Data are presented as the mean ± SEM. *P*-values < 0.05 were considered statistically significant.

## Supplementary information

original western blots

sw480 cell by STR profiling

sw620 cell by STR profiling

western blot original data and original images

Supplementary figure 1

Supplementary figure 2

Supplementary figure legends

## Data Availability

All data generated or analyzed during this study are included in this published article and Supplementary Materials.

## References

[1] Brenner H, Kloor M, Pox CP (2014). Colorectal cancer. Lancet.

[2] Dekker E, Tanis PJ, Vleugel JLA, Kasi PM, Wallace MB (2019). Colorectal cancer. Lancet.

[3] Mauri G, Sartore-Bianchi A, Russo AG, Marsoni S, Bardelli A, Siena S (2019). Early-onset colorectal cancer in young individuals. Mol Oncol.

[4] Read TE, Kodner IT (1999). Colorectal cancer: risk factors and recommendations for early detection. Am Fam Physician.

[5] Thanikachalam K, Khan G (2019). Colorectal cancer and nutrition. Nutrients.

[6] Fernandez E, La Vecchia C, D’Avanzo B, Negri E, Franceschi S (1997). Risk factors for colorectal cancer in subjects with family history of the disease. Br J Cancer.

[7] Keum N, Giovannucci E (2019). Global burden of colorectal cancer: emerging trends, risk factors and prevention strategies. Nat Rev Gastroenterol Hepatol.

[8] Kuipers EJ, Grady WM, Lieberman D, Seufferlein T, Sung JJ, Boelens PG (2015). Colorectal cancer. Nat Rev Dis Prim.

[9] Geng F, Wang Z, Yin H, Yu J, Cao B (2017). Molecular targeted drugs and treatment of colorectal cancer: recent progress and future perspectives. Cancer Biother Radiopharm.

[10] Schraivogel D, Weinmann L, Beier D, Tabatabai G, Eichner A, Zhu JY (2011). CAMTA1 is a novel tumour suppressor regulated by miR-9/9* in glioblastoma stem cells. EMBO J.

[11] Kim MY, Yim SH, Kwon MS, Kim TM, Shin SH, Kang HM (2006). Recurrent genomic alterations with impact on survival in colorectal cancer identified by genome-wide array comparative genomic hybridization. Gastroenterology.

[12] Lu H, Huan C (2007). Transcription factor NFAT, its role in cancer development, and as a potential target for chemoprevention. Curr Cancer Drug Targets.

[13] Pan MG, Xiong Y, Chen F (2013). NFAT gene family in inflammation and cancer. Curr Mol Med.

[14] Konig A, Fernandez-Zapico ME, Ellenrieder V (2010). Primers on molecular pathways–the NFAt transcription pathway in pancreatic cancer. Pancreatology.

[15] Imai Y, Maru Y, Tanaka J (2016). Action mechanisms of histone deacetylase inhibitors in the treatment of hematological malignancies. Cancer Sci.

[16] Sun X, Kaufman PD (2018). Ki-67: more than a proliferation marker. Chromosoma.

[17] Yang TTC, Xiong Q, Enslen H, Davis RJ, Chowi CW (2002). Phosphorylation of NFATc4 by p38 mitogen-activated protein kinases. Mol Cell Biol.

[18] Tang Q, Chen J, Di Z, Yuan W, Zhou Z, Liu Z (2020). TM4SF1 promotes EMT and cancer stemness via the Wnt/β-catenin/SOX2 pathway in colorectal cancer. J Exp Clin Cancer Res.

[19] Peng W, Li J, Chen R, Gu Q, Yang P, Qian W (2019). Upregulated METTL3 promotes metastasis of colorectal cancer via miR-1246/SPRED2/MAPK signaling pathway. J Exp Clin Cancer Res.

[20] Qin CJ, Bu PL, Zhang Q, Chen JT, Li QY, Liu JT (2019). ZNF281 regulates cell proliferation, migration and invasion in colorectal cancer through Wnt/β-catenin signaling. Cell Physiol Biochem.

[21] Tang W, Zhou W, Xiang L, Wu X, Zhang P, Wang J (2019). The p300/YY1/miR-500a-5p/HDAC2 signalling axis regulates cell proliferation in human colorectal cancer. Nat Commun.

[22] Schmittgen TD, Livak KJ (2008). Analyzing real-time PCR data by the comparative C_T_ method. Nat Protoc.

[23] Merten OW, Hebben M, Bovolenta C (2016). Production of lentiviral vectors. Mol Ther Methods Clin Dev.

