# Supplementary material for: CAMTA1–PPP3CA–NFATc4 multi-protein complex mediates the resistance of colorectal cancer to oxaliplatin
Source: Cell Death Discov. 2022 Mar 24;8:129. doi: 10.1038/s41420-022-00912-x (PMC8948201; doi:10.1038/s41420-022-00912-x)

# 细胞遗传质量鉴定检测

# Cell Line Authentication Service

## STR 基因型检测报告


电话：+86-400-650-3656
网址： www.procell.com.cn
Tel：+86-400-650-3656
Web：www.procell.com.cn

# 报 告 说 明

1. 本报告只对送检的来样负责。
2. 检验报告上的检验结果和检验单位名称，未经同意不得用于广告、评优及商业宣传。
3. 对本报告有异议，请于收到报告之日起十五日内以书面方式提出，逾期不予受理。
4. 对纸质检验报告涂改、增删，或未加盖检验单位印章的复印件均无效。


电话：+86-400-650-3656　　Tel：+86-400-650-3656
网址： www.procell.com.cn　　Web：www.procell.com.cn

# 样 品 信 息

**样品编号：**

| 客户样本编号 | 编号 |
|---|---|
| 07-06-1 | 01 |

**样品数量：** 1

**样品性状：** 细胞株

**检测项目：** STR

**检测方法：** 用 Axygen 的基因组抽提试剂盒提取 DNA，采用 21- STR 扩增方案扩增，在 ABI 3730XL 型遗传分析仪上对 STR 位点和性别基因 Amelogenin 进行检测。


电话：+86-400-650-3656
网址：www.procell.com.cn
Tel：+86-400-650-3656
Web：www.procell.com.cn

# 检 测 结 果

## (一) 检验基本情况

| 编号 | 匹配细胞系 | 细胞库 | EV 值 | 匹配说明 |
|---|---|---|---|---|
| 01 | SW480 | ATCC; CCL-228<br>DSMZ; ACC-313<br>ECACC; 87092801. | 0.9512 | 基本匹配 |

样本基因型检验结果

- 该细胞株 DNA 分型在细胞库中找到与其细胞分型 95.12%相匹配的细胞株，细胞株名称为 **SW480**。
- 本次检测各细胞分型结果良好。
- SW480 细胞株 DNA 进行细胞 STR 分型结果显示，细胞株中未发现人类细胞交叉感染。

## (二) 各样本描述

- 01：该株细胞 DNA 分型在细胞系检索中找到**基本匹配**的细胞系，数据库显示细胞名为 **SW480**。


电话：+86-400-650-3656 Tel：+86-400-650-3656
网址： www.procell.com.cn Web：www.procell.com.cn

## （三）样本分型结果

| | | | |
|---|---|---|---|
| Amelogenin | X | D21S11 | 30,30.2 |
| CSF1PO | 13,14 | FGA | 24 |
| D2S1338 | 17,24 | PentaD | 9,15 |
| D3S1358 | 15 | PentaE | 10 |
| D5S818 | 13 | TH01 | 8 |
| D7S820 | 8 | TPOX | 11 |
| D8S1179 | 13 | vWA | 16 |
| D13S317 | 12 | D1S1656 | 13,14 |
| D16S539 | 13 | D6S1043 | 11,12 |
| D18S51 | 13 | D12S391 | 17 |
| D19S433 | 13 | D21S11 | 30,30.2 |


电话：+86-400-650-3656 Tel：+86-400-650-3656
网址： www.procell.com.cn Web：www.procell.com.cn

## （四）SW480 细胞 STR 位点和 Amelogenin 位点的基因分型

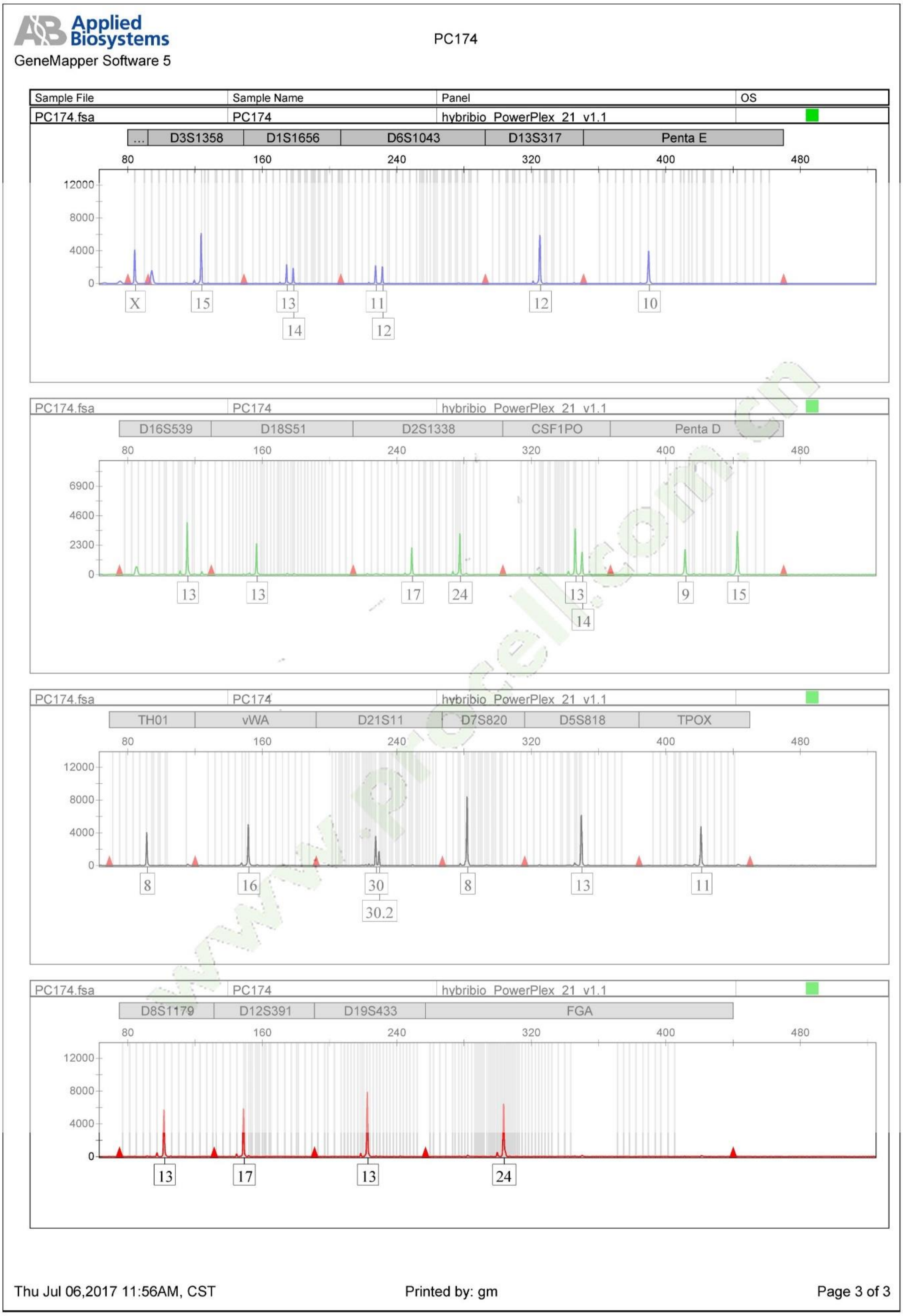

Supplement: Supplementary file 2 — sw480 cell by STR profiling [file 41420_2022_912_MOESM2_ESM.pdf]
