# Supplementary figures and images for: CAMTA1–PPP3CA–NFATc4 multi-protein complex mediates the resistance of colorectal cancer to oxaliplatin
Source: Cell Death Discov. 2022 Mar 24;8:129. doi: 10.1038/s41420-022-00912-x (PMC8948201; doi:10.1038/s41420-022-00912-x)

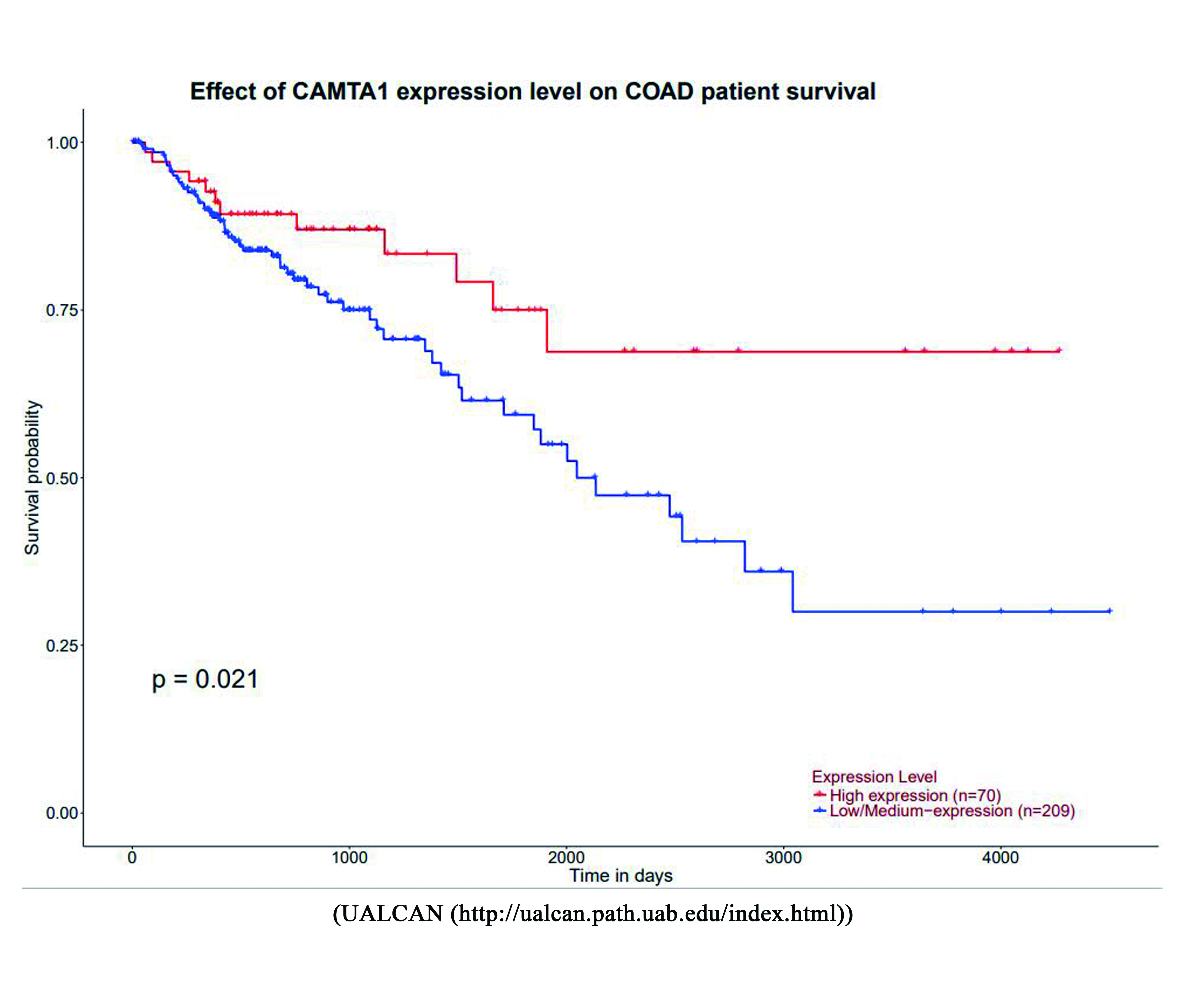

Supplement: Supplementary file 5 — Supplementary figure 1 [file 41420_2022_912_MOESM5_ESM.tif]

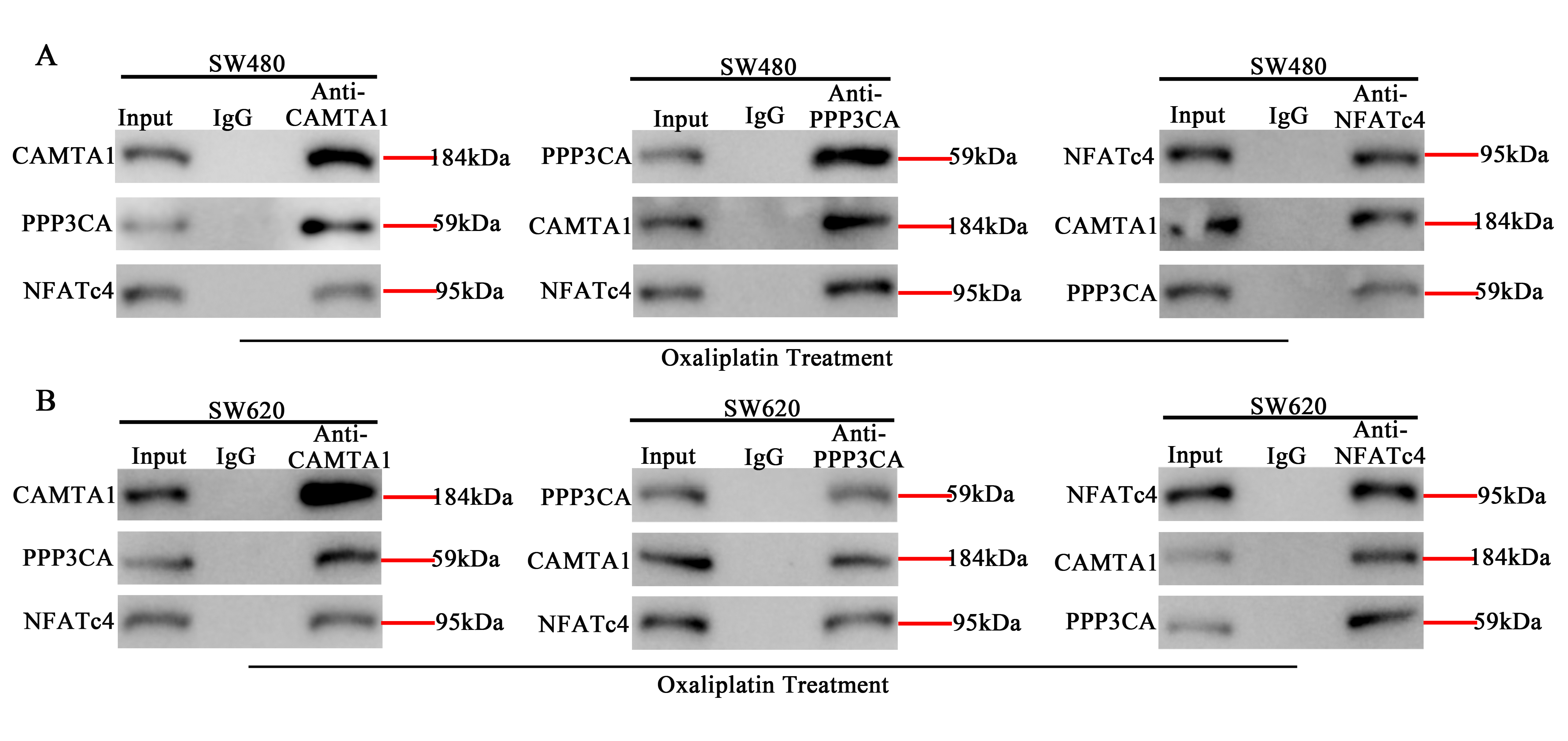

Supplement: Supplementary file 6 — Supplementary figure 2 [file 41420_2022_912_MOESM6_ESM.tif]
