# Supplementary material for: CAMTA1–PPP3CA–NFATc4 multi-protein complex mediates the resistance of colorectal cancer to oxaliplatin
Source: Cell Death Discov. 2022 Mar 24;8:129. doi: 10.1038/s41420-022-00912-x (PMC8948201; doi:10.1038/s41420-022-00912-x)
Supplement: Supplementary file 7 — Supplementary figure legends [file 41420_2022_912_MOESM7_ESM.docx]

**Supplementary figure legends**

**Supplementary figure 1.** The overall survival time of colorectal cancer patients with different expression level of CAMTA1 was determined by using the UALCAN web tool (<http://ualcan.path.uab.edu/index.html>), P values as indicated.

**Supplementary figure 2. Co-immunoprecipitation and western blotting analyses of interactions among CAMTA1, NFATC4, and PPP3CA in SW480 and SW620 cells with the treatment of oxaliplatin.** (A) Co-immunoprecipitation and western blotting analyses of interactions among CAMTA1, NFATC4, and PPP3CA in SW480 cells with the treatment of oxaliplatin. (B) Co-immunoprecipitation and western blotting analyses of interactions among CAMTA1, NFATC4, and PPP3CA in SW620 cells with the treatment of oxaliplatin. Input IgG served as the internal control.
